# Ultrasound-Guided Erector Spinae Plane Block in Emergency Department for Abdominal Malignancy Pain: A Case Report

**DOI:** 10.5811/cpcem.2022.3.55752

**Published:** 2022-11-05

**Authors:** Henry Ashworth, Noah Sanders, Daniel Mantuani, Arun Nagdev

**Affiliations:** Alameda Health System, Highland Hospital, Department of Emergency Medicine, Oakland, California

**Keywords:** ultrasound-guided nerve block, erector spinae plane block, breakthrough pain, cancer pain, case report

## Abstract

**Introduction:**

Severe breakthrough pain is a common occurrence in patients with cancer and is responsible for thousands of emergency department (ED) visits each year. While opioids are the current mainstay of treatment, they have multiple limitations including inadequate control for a quarter of patients with cancer. The ultrasound-guided erector spinae plane block (ESPB) has been used in the ED to effectively treat pain for pathologies such as acute pancreatitis, since it provides somatic and visceral analgesia.

**Case Report:**

In this case report we describe the use of an ESPB to treat breakthrough pain safely and effectively in a 54-year-old female with a history of metastatic colon cancer.

**Conclusion:**

The ESPB may have utility in addressing well documented disparities in pain treatment in the ED, but additional research is needed to understand side effects, duration of pain control, and clinical outcomes of the ESPB.

## INTRODUCTION

Severe breakthrough pain is a common occurrence in patients with cancer, leading to thousands of emergency department (ED) visits for symptom control.^1^,^2^ Patients with chronic abdominal pain from cancer are particularly susceptible to breakthrough pain, with over half experiencing severe breakthrough pain episodes.^2^

Opioids are the mainstay of treatment for breakthrough pain. Unfortunately, opioids have several limitations and adverse effects. First, they do not provide relief for up to a quarter of chronic cancer pain patients.^3^ Second, opioids can cause respiratory depression, delirium, hypotension, and constipation. Third, patients often develop tolerance with long-term use.^4^ Finally, when the primary oncology or pain team cannot be reached, many emergency physicians may be hesitant to add additional opioids to a patient’s current regimen. Overall, research is limited on how to effectively provide non-opioid treatment for acute and breakthrough cancer pain in the ED.

Nerve blocks, a mainstay in the World Health Organization analgesic ladder for pain management, offer a short-term analgesic solution without the side effects of opioids.^4^ In acute on chronic pain treatment, ultrasound-guided nerve blocks may also break the “wind-up phenomenon” where severe pain is felt when a stimulus is repeated above a critical rate.^5^ Blocking this hyperexcitable, central sensitization in chronic and acute pain patients may allow for improved longer term pain control. The ultrasound-guided erector spinae plane block (ESPB) has been shown to effectively treat pain in the ED for a number of pathologies including acute pancreatitis, appendicitis, and rib fractures.^6^,^7^ The ESPB is especially useful in treating abdominal pain as it provides somatic analgesia at the dorsal and ventral rami of the spinal nerves, and visceral analgesia by blocking the sympathetic fibers.^8^ Here we present the first description of a lower thoracic ESPB to successfully manage breakthrough pain due to an abdominal malignancy in the ED.

## CASE REPORT

A 54-year-old female with a history of metastatic colon cancer presented to the ED with persistent right-sided abdominal pain refractory to her prescribed home pain regimen. The patient had numerous recent ED visits and admissions for persistent abdominal pain. She had been placed on methadone 35 milligrams (mg) daily for opiod use disorder yet continued to have breakthrough pain after a prescribed oral protocol (from the outpatient pain management service) of hydromorphone 4 mg every six hours in conjunction with gabapentin 300 mg every eight hours. All her other labs were similar to her previous visit five days earlier except for an elevated alkaline phosphatase of 490 units per liter (U/L), which trended up from 275 U/L (reference range: 44–147 U/L). Three days prior to the ED visit, a computed tomography of the abdomen had shown numerous hepatic metastatic lesions. After four intravenous doses of 8 mg of morphine and one intravenous dose of 25 mg of ketamine (over a three-hour ED visit), the patient’s pain persisted.

The emergency medicine team planned for an inpatient admission for persistent, intractable abdominal pain. Because of the patient’s severe and refractory pain, a right-sided ultrasound-guided ESPB was offered to her. After consenting her for the procedure and placing her on a cardiac monitor, a 13–6 megahertz linear transducer was placed longitudinally and paraspinal on the right lower thorax. The patient remained in a seated position at the edge of the bed, and ultrasound was used to locate the transverse process at the ninth thoracic vertebra (T9). The area was disinfected with chlorhexidine, and a wheal of local anesthesia was placed. A 21-gauge × 100-millimeter SonoBlock II Facet S block needle (The Pajunk Group, Geisingen, Baden-Württemberg, Germany) was advanced in-plane from cranial to caudal direction until the needle tip touched the superior portion of the transverse process of the T9 (Image).

To ensure correct placement of local anesthetic, 10 milliliters (mL) of normal saline was used to hydrodissect the dorsal fascial plane of the erector spinae muscle overlying the transverse process. After negative aspiration, 20 mL of 0.5% bupivicaine was gradually injected in 3–5 mL aliquots. After the anesthetic was gradually placed in the fascial plane below the erector spinae muscle, 10 mL of normal saline was again injected to flush the line as well as dilute the anesthetic.

Thirty minutes after the ESPB was performed the patient reported complete resolution of her pain. She was able to sleep comfortably and reported 1/10 pain when awakened by the admitting medicine attending. The patient was evaluated by the inpatient internal medicine service who contacted her oncologist. She was discharged with an increased oral pain protocol and a 24-hour follow-up with her oncologist. There were no reported complications from the ESPB.

**CPC-EM Capsule**

What do we already know about this clinical entity?

*This is the first reported case of the erector spinae plane block being used in the emergency department for breakthrough chronic pain from malignancy*.

What makes this presentation of disease reportable?

*The erector spinae plane block is a safe and effective block that can be utilized for acute on chronic abdominal pain in the emergency department (ED)*.

What is the major learning point?

*By providing effective pain management without serious side effects to the thousands of chronic pain patients who visit the ED annually*.

How might this improve emergency medicine practice?

*The erector spinae plane block can offer safe and effective treatment of somatic and visceral pain for patients in the emergency department*.

## DISCUSSION

This case adds to the body of literature that indicates ESPB is safe, simple, and efficacious in treating thoracoabdominal pain in the ED.^6^,^7^ The ESPB is uniquely effective as it can treat both somatic and visceral pain without the harmful side effects of opioids.^3^,^8^–^10^ To our knowledge, this is the first case of an ESPB for the treatment of acute on chronic abdominal pain due to metastatic abdominal disease in the ED. As abdominal pain from metastatic disease is a common reason why patients present to the ED, this block offers a promising solution to treat their pain.^11^

The ESPB may have utility in addressing several well documented disparities in pain treatment in the ED. As already discussed, ESPB could have the ability to provide adequate pain relief for under-treated pain from cancer.^10^ An aversion to prescribing opioids and the evidence of racial bias in prescribing patterns, as well as a lack of pain management specialists, have led to poor treatment of acute pain in all patients, but particularly in racial minorities.^10^,^12^ Patients of color experience great disparities in opioid prescriptions in the ED due to racial bias and the social stigma related to opioid use.^13^,^14^ In the ED, Black patients are prescribed opioids less often than Whites across all levels of socioeconomic status.^13^ Addressing this disparity in treatment has no simple solution and requires addressing systemic, interpersonal, and internalized discrimination. Removing the social stigma and bias related to opioids with increased utilization of blocks such as the ESPB could be a singular step in improving equitable and more effective treatment in the ED.

While our group has not experienced any complications related to the ESPB (such as pneumothorax, anesthetic systemic toxicity, or lower extremity weakness) complication rates in the ED should be explored. The impacts of increasing use of ultrasound-guided nerve blocks for pain management in the ED could parallel outcomes seen in the inpatient setting such as reduced opioid use, decreased admission time, lower cost to patients, and improved recovery.^15^ As the evidence continues to grow for the successful use of ESPB in the ED for severe pain, there is a need for more systematic studies to investigate side effects, duration of pain control, and clinical outcomes.

## CONCLUSION

As EDs continue to face challenges in adequately treating patients with acute on chronic pain from cancer, nerve blocks such as the ESPB offer a solution. Blocks such as the ESPB also offer several advantages over opioids and could lead to more equitable treatment of pain. While this case report adds to the growing levels of evidence that the ESPB may help treat acute pain for abdominal pathologies in the ED, more research is needed to understand the large-scale efficacy and adverse effects of the ESPB.

## Figures and Tables

**Image:**
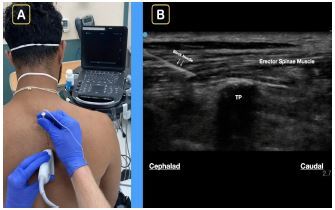
A) A representative patient is in seated position with the ultrasound screen in clear line of sight. A blunt-tip block needle is used in-plane cranial to caudal. B) At approximately the T9 level, note the block needle clearly visualized and aiming for the transverse process (TP). The goal is to spread anesthetic under the erector spinae muscle.
